# Enhancing Outcomes of Low-Intensity Parenting Groups Through Sufficient Exemplar Training: A Randomized Control Trial

**DOI:** 10.1007/s10578-018-0847-z

**Published:** 2018-10-09

**Authors:** Melanie L. Palmer, Louise J. Keown, Matthew R. Sanders, Marion Henderson

**Affiliations:** 1 Faculty of Education and Social Work, School of Learning, Development and Professional Practice, The University of Auckland, Symonds St, Private Bag 92601, Auckland, 1142 New Zealand; 2 Medical Research Council/Chief Scientist Office Social and Public Health Sciences Unit, University of Glasgow, 200 Renfield Street, Glasgow, G2 3QB UK; 3 Parenting and Family Support Centre, The University of Queensland, Brisbane, QLD 4072 Australia

**Keywords:** Parenting, Intervention, Low-intensity, Conduct problems, Generalization

## Abstract

Low-intensity parenting groups, such as the Triple P-Positive Parenting Program Discussion Groups, appear to be a cost-effective intervention for child conduct problems. Several studies evaluating a Triple P Discussion Group on disobedience found promising results for improving child and parent outcomes. However, a sufficient exemplar training approach that incorporates generalization promotion strategies may assist parents to more flexibly apply positive parenting principles to a broader range of child target behaviors and settings, leading to greater change. We compared the effects of sufficient exemplar training to an existing narrowly focused low-intensity intervention. Participants were 78 families with a 5–8 year-old child. Sufficient exemplar training resulted in more robust changes in child behavior and superior outcomes for mothers on measures of parenting behavior, parenting self-efficacy, mental health, and perceptions of partner support at post-intervention and 6-month follow-up. These results indicate that teaching sufficient exemplars may promote generalization leading to enhanced intervention outcomes.

## Introduction

There is a high prevalence of mild to moderate levels of conduct problems displayed by young children in the population with estimates ranging from 19 to 65% [1, 2]. Costs associated with child conduct problems include stress and concern for the child and their caregivers [2, 3], poorer short- and long-term academic, peer, health, and behavioral outcomes [4, 5] and demands on public services [6]. Survey data also indicate a high prevalence of ineffective parenting practices among parents of young children with between 10–70% reporting that they shout at or use physical punishment with their child [2, 7, 8] highlighting a need for effective interventions. Although there appears to be a substantial proportion of families who would benefit from a parenting program, participation is low [2]. A possible reason for low participation in parenting programs is that available programs are not meeting the needs or preferences of all parents. For some families, an 8–18 week intensive program may be required, however in other families a long-term intervention may be neither feasible nor needed [9].

Low-intensity parenting programs play an important role in a public health approach to parenting support that aims to reduce the prevalence of child conduct problems at a population level [10]. Such programs require less practitioner time, are more cost-effective [11] and range from single session programs to several sessions of topic-focused intervention [9]. Typically, low-intensity parenting programs focus on a narrow range of specific child problems or parenting strategies. For parents of a child with mild to moderate conduct problems, a low-intensity program may be sufficient to prevent the development of more serious problems [12]. There is evidence that low-intensity parenting programs lead to positive changes in children and parents. A recent systematic review of low-intensity parenting programs, delivered individually and in group settings, reported reductions in disruptive child behavior and ineffective parenting behaviors, as well as improvements in parenting self-efficacy and satisfaction at post-intervention [13].

The potential benefits of low intensity interventions could be further enhanced by building in strategies that promote generalization, transfer of learning, and psychological flexibility effects. One strategy to promote more flexible application of parenting skills that is particularly well suited to the delivery of low-intensity topic-specific parenting groups is teaching a sufficient number of training exemplars. Teaching a sufficient number of exemplars involves providing enough examples and illustrations of how to apply positive parenting and contingency management principles so that the transfer of skills across diverse contexts is promoted [14]. Single exemplar training may result in change of the exemplar taught, but more limited generalization to other child or parent behaviors or settings. For example, a low-intensity parenting program that teaches parents skills to manage specific forms of misbehavior (e.g., disobedience) may not be sufficient for parents to generalise parenting skills to manage other difficult child behaviors such as aggression, or to manage difficult behaviors in a range of settings. Teaching a second or third exemplar may facilitate the spread of intervention effects across a broader range of child and parent outcomes. Sufficient exemplar training (SET) also incorporates other generalization promotion strategies, such as training loosely [14] to enhance parents’ ability to flexibly apply positive parenting and behavior management principles skills across a broader range of contexts (behaviors and settings) leading to more robust changes across a diverse range of child and parent outcomes.

The principle of teaching sufficient exemplars can be readily applied to the Triple P Discussion Groups (TPDG) [15], which are low-intensity parenting programs. It would involve parents attending a series of topic-specific low-intensity parenting groups, where core parenting principles and skills are taught using a diverse range of exemplar topics. Those who receive teaching in sufficient exemplars learn how core behavioral parenting strategies (e.g., praise, applying logical consequences) are applied to a range of specific behaviors (e.g., disobedience, fighting and aggression, chores, self-esteem).

In the current study, SET comprised participating in four TPDGs, which are 2-hour group interventions that teach parents skills to manage a specific child behavior problem or developmental issue. Each group session introduced core parenting skills and principles including application of anticipatory antecedent events (e.g., discussion of rules, planned activities, giving clear instructions) and consequent events (e.g., praise, logical consequences) and their application to a diverse range of topics that include increasing prosocial behaviors, building resilience and reducing problem behaviors. We predicted that this kind of training would help consolidate the learning of these skills and result in more robust intervention effects across a broader range of child and parent outcomes. We also predicted that SET would lead to more robust changes at multiple levels of the family system, such as parental mental health and their partner relationship, than narrowly focused training.

In testing this new intervention, we wanted to benchmark its effects against an existing evidence-based low-intensity program, the Triple P Dealing with Disobedience Discussion Group (DDDG). We chose this low-intensity program because it has an established evidence base, is widely disseminated, has high levels of consumer satisfaction, could go some way to control for expectancy effects associated with receiving an evidence-based intervention, and would avoid the ethical concern of having parents wait to receive an intervention. In choosing this single session intervention as a comparator condition we were mindful of the fact that the two conditions had differing amounts of contact time, albeit a relatively small difference of 2 versus 8 hours. Both are low-intensity interventions in the parent training field. Three evaluations using randomized control trial (RCT) designs have found that following DDDG parents report significant reductions in child behavior problems and less use of ineffective parenting practices at post-intervention [16–18] in comparison to a waitlist control condition and that effects are maintained at 6-month follow-up. Significant reductions in parenting self-efficacy, poor parental mental health and inter-parental conflict have also been found at 6-month follow-up [16, 18]. However, a lack of follow-up data for the waitlist control group limits conclusions. In Mejia et al. [17], follow-up data was obtained from both groups at follow-up and effects on parenting practices and mental health were found. High levels of satisfaction with the DDDG have also been reported.

This study also targeted a research gap on the effects of low-intensity parenting programs for fathers [13, 19], by attempting to engage fathers as well as mothers in the study. We aimed to explore the extent to which fathers’ participation has similar outcomes to mothers’ participation on child and parenting outcomes. An additional focus of the study was to examine the effects of the Triple P Discussion Groups among parents with young school-aged children (defined in this study as 5–8 year olds) by addressing key topics relevant to this developmental phase, including fighting and aggression, chores, and self-esteem. This emphasis is important given key changes in parenting tasks during middle childhood, which relate to an increase in children’s regulation of their own behavior and an increase in interactions with others (e.g., non-familial adults, peers [20]). For parents, the increasing number of external influences on their children’s development may result in changes in parental monitoring and create new challenges around promoting positive development (e.g., getting along with peers at school). In contrast, the previous research evaluating the DDDG has used samples of parents with preschool aged children.

## Method

### Trial Registration

The trial was registered on the Australian New Zealand Clinical Trials Registry (Reference ACTRN12613000100796).

### Participants

Participants were 75 mothers and 58 fathers from 78 families with a 5–8 year old child residing in Auckland, New Zealand (see Table 1 for the demographic characteristics of the participants). The majority of families in the sample were two-parent families (84.6%, *n* = 66). There were 55 mother-father pairs from the same family, 20 mothers participated alone (nine of which were from two-parent families), and three fathers participated alone (two of which were from two-parent families). The majority of the target children were male (*n* = 50) and of New Zealand European/New Zealander ethnicity (71.8%, *n* = 56). A high proportion of the families reported that their total family income was greater than $50,000 per annum. Many mothers and fathers in the current sample had a university degree (56.8% and 49.1% respectively). All fathers, except one, and about two-thirds of mothers were in paid employment. There were no significant differences between the two conditions on any demographic variable or pre-intervention measures. Thus, randomization to condition resulted in two groups that were similar at pre-intervention.

**Table 1:** Demographic details of participating families by condition

Variable	DDDG Families: *N* = 35 Mothers: *N* = 34 Fathers: *N* = 27		SET Families: *N* = 43 Mothers: *N* = 41 Fathers: *N* = 31	
	*n*	%	*n*	%
Child gender				
Male	23	65.7	27	62.8
Female	12	34.3	16	37.2
Child ethnicity				
New Zealand European/New Zealander	28	80.0	28	65.1
Maori	1	2.9	0	0.0
Pacific Islander	1	2.9	1	2.3
Asian	1	2.9	3	7.0
Other	4	11.4	11	25.6
Type of family				
Two-parent biological or adoptive	26	74.3	31	72.1
Two-parent step family	2	5.7	7	16.3
Single parent family	7	20.0	5	11.6
Marital status				
Married	22	62.9	29	67.4
Cohabiting	6	17.1	9	20.9
Divorced	0	0.0	1	2.3
Separated	6	17.1	3	7.0
Single	1	2.9	1	2.3
Total family income				
< $30,000	3	8.6	5	11.6
$30,001–$50,000	5	14.3	6	14.0
$50,000–$70–75,000	2	5.7	7	16.3
> $70–75,000	25	71.4	23	53.5
Don’t know	0	0.0	2	4.7
Mother relationship to child				
Biological or adoptive parent	34	100.0	41	100.0
Father relationship to child				
Biological or adoptive parent	26	96.3	26	83.9
Step-parent	1	3.7	5	16.1
Mother highest level of education*				
Year 13 or less	4	12.1	8	19.5
Polytechnic qualification	6	18.2	11	26.8
Trade/apprenticeship	2	6.1	1	2.4
University degree	21	63.6	21	51.2
Father highest level of education*				
Year 13 or less	5	19.2	7	22.6
Polytechnic qualification	5	19.2	6	19.4
Trade/apprenticeship	4	15.4	2	6.5
University degree	12	46.2	16	51.6
Mother in paid employment	22	64.7	25	61.0
Father in paid employment	26	96.3	31	100.0

	M	SD	M	SD
Mother age	38.41	4.66	37.52	5.03
Father age	40.22	4.55	39.55	5.77
Mothers hours in paid employment^a^	30.19	13.22	30.34	13.91
Fathers hours in paid employment^b^	43.17	8.74	41.71	7.33

*Data was missing for one participant, valid % reported

^a^*n* = 18 for DDDG condition, *n* = 22 for SET condition

^b^*n* = 18 for DDDG condition, *n* = 26 for SET condition

### Measures

#### Demographics

The Family Background Questionnaire [21] was used to obtain demographic characteristics of participants at pre-intervention.

#### Child Outcomes

The primary outcome measure was the Eyberg Child Behavior Inventory (ECBI) [22]. The ECBI is a 36-item questionnaire that measures parents’ perceptions about the frequency (Intensity subscale) and number (Problem subscale) of disruptive child behaviors. Responses are summed to create a total score for each subscale. Scores on the ECBI Intensity subscale range from 36 to 252 and scores on the Problem subscale range from 0 to 36. ECBI Intensity Total scores equal to or greater than 131 and ECBI Problem Total scores equal to or greater than 15 indicate clinically elevated disruptive behavior problems. Most mothers reported clinically elevated scores on the ECBI Intensity and Problem subscales at pre-intervention (69.3% and 82.7% respectively). Among fathers, 60.3% and 65.5% reported clinically elevated scores on these subscales respectively. Internal consistency was high at all time points for both subscales among mothers (*α* = 0.79–0.93) and fathers (*α* = 0.87–0.92).

The Strengths and Difficulties Questionnaire (SDQ) [23] is a 25-item questionnaire that measures parents’ perceptions of their child’s hyperactivity, conduct, emotional, and peer problems, and prosocial behavior. Responses on all subscales except the prosocial behavior subscale are summed to obtain a SDQ Total Difficulties score (possible range 0–40). Cronbach’s alphas were adequate at all time points for mothers (0.77–0.86) and fathers (0.72–0.79).

The Parent Daily Report Checklist (PDR) [24] asks parents to report the occurrence of a range of child behaviors for a weekday and a weekend day. It provided a measure of change in target and non-target negative child behaviors. Three items were added to the existing 25 items to measure problems with chores (e.g., refusing to do chores or jobs). Total scores were calculated to produce a total number of negative behaviors displayed for each particular day and range from 0 to 28. Internal consistency was adequate for both subscales among mothers and fathers across all time points (*α*’s were 0.72–0.87 and 0.73–0.88 respectively).

#### Parenting Outcomes

The Parenting Scale (PS) [25] is a 30-item questionnaire measuring the use of ineffective and negative parenting behaviors. Scores range from 1 to 7 with higher scores indicating more ineffective parenting behaviors. The PS demonstrated good internal consistency at all time points for mothers (*α* = 0.84–0.88) and fathers (*α* = 0.86–0.92).

The Parenting Tasks Checklist (PTC) [26] is a 28-item measure that taps into parents’ self-efficacy for handling difficult child behaviors (behavioral) and confidence in dealing with misbehavior in different settings (setting). Responses to the items are averaged to produce total behavioral and setting parenting self-efficacy scores (possible range 0–100). Internal consistency was high for both subscales among mothers and fathers across all time points (*α*’s were 0.92–0.99 and 0.87–0.97 respectively).

The Parenting Experience Survey Parenting Experience subscale (PES Parenting Experience) [21] was used to measure perceptions of the parents’ experience in their parenting role. A total score is calculated by summing the responses to the 5 items with a possible range of 5–25. Higher scores indicate a more positive parenting experience. Cronbach’s alphas were adequate at all time points for mothers (0.73–0.78) and fathers (0.58–0.78).

The Parent Problem Checklist (PPC) [27] was used to measure the extent (Extent subscale) and number (Problem subscale) of child-rearing disagreements between parents among two-parent families. The PPC is made up of 16 items. Responses are summed to produce total scores. Scores on the Extent and Problem subscales range from 16 to 112 and 0 to 16 respectively. Internal consistency was good at all time points for both subscales among mothers (*α* = 0.83–0.95) and fathers (*α* = 0.81–0.94).

#### Mental Health

The Depression Anxiety Stress Scales 21-item version (DASS-21) [28] measured symptoms of depression, anxiety and stress experienced by parents. A DASS-21 Total score is calculated by summing responses to the 21 items (range 0–63). High internal consistency was found for mothers and fathers at all time points (*α*’s were 0.87–0.91 and 0.89–0.93 respectively).

#### Partner Relationship

Parents from two-parent families completed the Parenting Experience Survey Partner Support subscale (PES Partner Support) [21] which measures perceptions of support from their partner. Responses to the 3 items are summed to produce a Partner Support Total score. Higher scores indicate greater partner support and range from 2 to 16. Cronbach’s alphas were adequate at all time points for mothers (*α* = 0.76–0.84) and fathers (*α* = 0.66–0.75).

The Relationship Quality Index (RQI) [29] measured partner relationship satisfaction among two-parent families. Responses to the 7 RQI items are summed with higher scores indicating greater satisfaction (possible range 7–45). Internal consistency was high for mothers and fathers at all time points (*α*’s were 0.95–0.97 and 0.93–0.96 respectively).

#### Participant Satisfaction

Participant satisfaction with and acceptability of each Triple P Discussion Group was measured using the Discussion Group Satisfaction Questionnaire (DGSQ) [21]. The DGSQ consists of 11 items, 10 of which are rated on a scale resulting in a possible range of 10 to 70. The final question asks for additional comments. The DGSQ was completed anonymously by attendees at the end of each group session. The Cronbach alpha for the DGSQs was 0.92.

### Design

A 2 (condition: DDDG vs. SET) by 3 (time: pre-intervention, post-intervention, 6-month follow-up) RCT design was used to compare the effects of the conditions on the outcome measures described above.

### Procedure

Ethical Approval was granted for the study by The University of Auckland’s Human Participants Ethics Committee (Reference 2011/360 and 7431). Advertising material was developed and disseminated in Central and West Auckland, New Zealand through a number of community outreach methods (e.g., local primary schools, press releases, local newspapers). The advertisement encouraged parents to self-refer to take part via email, phone, or text if they had a 5–8 year-old child who was showing some difficulties with their behavior and they were interested in attending a free brief discussion group-based parenting program. Participants were recruited between August and October in 2011 and February and May in 2014.

Upon contact, parents were informed of the study protocol and if interested screened for eligibility using an abbreviated 15 item version of the ECBI Intensity subscale [30]. Eligibility criteria included having a 5–8 year-old child displaying at least a mild level of conduct problems (a score of 45 or more on the ECBI screener). Potential participants were excluded from the study if: (1) the target child had a developmental or intellectual disability or other significant health impairment; (2) the target child was having regular contact with a health professional for behavioral problems or the parent was receiving support for the target child’s behavior problems; and (3) the parent was currently seeing a mental health professional for emotional or psychological problems. Families who did not meet the eligibility criteria were offered referral information for alternative services.

**Fig. 1:**
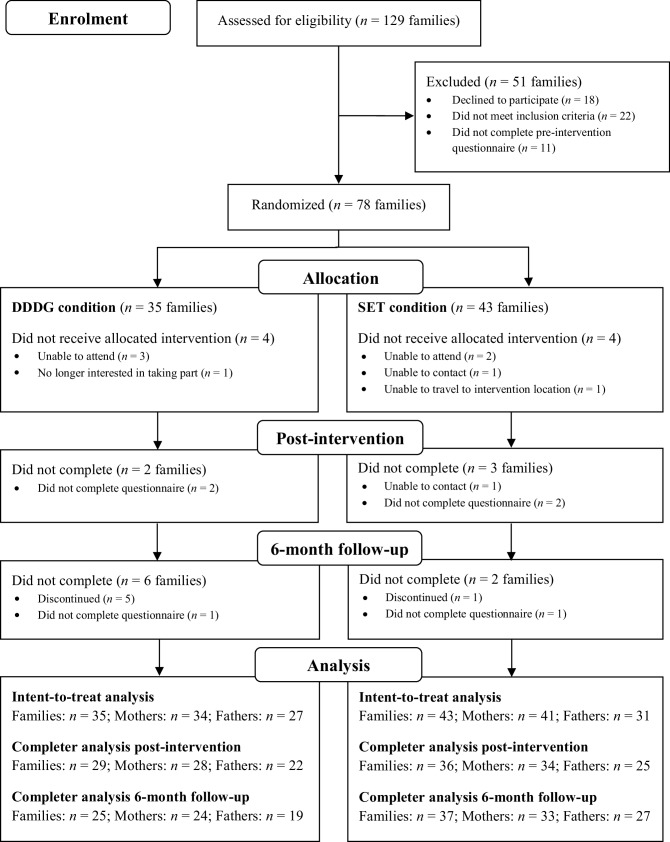
Flow of participants through each stage of the study and reasons for discontinuation

Eligible participants were enrolled in the study and sent a copy of the participant information sheet, the consent form, and the pre-intervention measures. After pre-intervention measures were completed, participants were randomly assigned to one of the two intervention conditions. Post-intervention measures were administered immediately after the end of the intervention for families allocated to the SET condition. For those in the DDDG condition, post-intervention measures were administered at the equivalent time of post-intervention for the SET condition (approximately 4 weeks after the intervention). Follow-up measures were administered approximately 6-months after post-intervention measures. Figure 1 displays the flow of participants through each stage of the study. The reporting of this study follows the CONSORT statement.

### Randomization

Randomization occurred at the level of individual families. Computer generated lists of random numbers were used to allocate to condition in sequence of completion of pre-intervention measures. In two-parent families where both parents consented to participating, allocation occurred when both parents had completed pre-intervention measures. In order to reduce the impact of any potential imbalances in allocation to condition, assignment was stratified by the area of Auckland participants resided in (Central or West) and household configuration (single-parent vs. two-parent household). To ensure there was no bias in allocation, randomization to condition was conducted by individuals independent of the study.

### Interventions

The Triple P Discussion Groups are 2-hour group interventions that teach parents skills to manage a specific child behavior problem or developmental issue. The strategies presented are alternatives to coercive and ineffective parenting behaviors and are tailored to focus on the specific topic that is being addressed. The information is presented in a variety of ways: parents watch video-modelling of strategies, complete within session exercises, and are given the opportunity to practice their skills in session, discuss the strategies with other group members and to formulate a parenting plan.

#### Narrowly Focused Training Condition, the Triple P Dealing with Disobedience Discussion Group (DDDG)

Parents in the single exemplar condition attended one TPDG on Dealing with Disobedience. The group covered reasons for disobedience and taught skills to encourage cooperation with parental instructions and to manage disobedience (e.g., logical consequences, quiet time, and timeout).

#### Sufficient Exemplar Training (SET) Condition

Parents allocated to the SET condition attended four TPDGs. There were two compulsory topics (Being a Positive Parent and Dealing with Disobedience) that all parents were asked to attend. Families were then asked to attend two additional sessions and could choose from three options targeting other specific behaviors and developmental concerns (Fighting and Aggression, Doing Chores, and Building Self-esteem). The Being a Positive Parent group introduced the principles of positive parenting and taught skills to enhance children’s competence and development and skills to build positive parent–child relationships. During the Fighting and Aggression group parents learnt about reasons for fighting and aggression and were taught strategies to teach children to play cooperatively and for dealing with sibling conflict, fighting, not sharing, and aggression. The Doing Chores group covered why helping out is important and taught skills to help prepare and encourage children to do chores as well as strategies for dealing with problems with chores. Parents who attended the Building Self-esteem group were taught the causes for low-self-esteem and ways to encourage healthy self-esteem and help children manage negative self-talk and solve problems. Parents allocated to the SET condition attended the Dealing with Disobedience and Being a Positive Parent groups before attending sessions on the additional topics. The groups were held weekly at the same time; thus, attendance at four groups occurred over a 4 or 5-week period depending on which additional topics parents chose.

It is important to note that those in the SET condition did not receive four times the amount of information or content. Rather core behavioral parenting strategies (e.g., praise, applying logical consequences) were taught in each session and applied to that specific behaviour. For example, using praise to increase desirable behavior focused on promoting compliance during the Dealing with Disobedience session, whereas during the Doing Chores session praise was applied to promoting completion of chores.

### Intervention Delivery

Twenty-one TPDGs were run on weekday evenings and the average group size was 11 parents (range 3–19 parents). All groups were delivered by the first author, who is a trained accredited Triple P Discussion Group practitioner, according to a standard manualised protocol [21]. Checklists created by the intervention developers were used to monitor intervention fidelity and provided a measure of the proportion of content covered in each group. Adherence to the intervention protocol was high (*M* = 92.5%, *SD* = 0.04). Inter-rater agreement between the adherence ratings provided by the practitioner and those provided by a second independent rater, who was a trained accredited Triple P practitioner, were high (*M* = 92.0%, *SD* = 0.04). Intervention fidelity ratings were similar across topics, therefore, there was no reason to believe that the groups in one condition were delivered with greater adherence to protocol than the other condition.

### Data Analysis

#### Power Calculation

The ECBI Intensity subscale was used to determine the sample size. Previous research [18, 31] from samples of primary caregivers, mainly mothers, was used to estimate the expected mean and standard deviation at pre-intervention (the average of the two studies was: *M* = 134, *SD* = 22). These studies reported that the effect size for disruptive child behavior following a single Triple P Discussion Group was medium to large. For a medium difference in effect sizes (*d* = 0.50) [32] at post-intervention between the two conditions, assuming a standard deviation of 22 (thus an estimated 11-point difference in ECBI Intensity Total scores), 64 families per condition would be required to achieve power of 80% at an alpha of 0.05. Post-hoc power calculations indicated that the observed power for the difference in ECBI Intensity Total scores at post-intervention was 93% for mothers.

#### Statistical Analyses

An intent-to-treat (ITT) analysis was used to examine the effects of the two conditions by the original assigned groups. Little’s Missing Completely at Random examining the pattern and extent of missing data indicated that multiple imputation (MI) was appropriate. Missing items were imputed five times using the predictive mean matching method. As the patterns of data were likely to differ according to intervention condition and parent gender, missing data were imputed separately for mothers and fathers in each condition.

To examine the short- and long-term condition effects of the two interventions, MANCOVAs and ANCOVAs were conducted using pre-intervention scores as covariates and either post-intervention or 6-month follow-up scores as dependent variables [33]. MANCOVA was used for conceptually related dependent variables (ECBI, PDR, PTC, PPC) and ANCOVA was used for unidimensional measures (SDQ, PS, PES Parenting Experience, DASS, PES Partner Support, RQI). Where there was the presence of multicollinearity between variables on a MANCOVA, ANCOVAs were conducted on each of these subscales instead. Effect sizes were calculated to determine the effect of the SET condition over the DDDG condition. These were calculated by subtracting the change from pre- to post-intervention in the DDDG condition from the change in pre- to post-intervention in the SET condition and dividing this by the pooled pre-intervention standard deviation [34]. A similar procedure was used to calculate effect sizes from pre-intervention to 6-month follow-up. Ninety-five percent confidence intervals were calculated on the pre- to post-intervention and pre-intervention to 6-month follow-up effect sizes.

The Reliable Change Index (RCI) [35] and the clinical cut-offs were used to examine statistically reliable and clinically significant change from pre- to post-intervention on the ECBI for each condition. Chi-squared tests were used to examine differences in distribution between the two conditions. Fisher’s exact χ^2^ tests were used as there were fewer than five cases in some categories.

## Results

### Attrition

Attrition in the current study was relatively low (see Fig. 1 for flow of participants through each stage of the study). Preliminary analyses revealed some statistically significant differences in family demographics, parental demographics, and pre-intervention measures between those who completed and those who did not complete outcome measures at post-intervention and 6-month follow-up. Among families who did not complete post-intervention measures, there were more cohabiting and separated families, more step- and single-parent families, and more families in the bottom family income range (< $30,000 per annum). There was also an over-representation of mothers with low levels of education. The only significant difference between families, mothers, and fathers who completed 6-month follow-up measures and those that did not was that fathers who completed 6-month follow-up measures had significantly higher PPC Problem Total scores at pre-intervention indicating greater inter-parental conflict prior to intervention.

### Attendance

Generally, attendance was high with the majority of families in the DDDG condition attending the session (88.6%), and a large portion of families in the SET condition attending two or more of the four sessions (81.4%). More mothers in the DDDG condition (85.3%) attended the session than fathers (59.3%). For those in the SET condition, a similar proportion of mothers and fathers attended two or more sessions (73.1% and 67.8% respectively), although more fathers did not attend any sessions (25.8%) when compared to mothers (14.6%). Among two-parent families, many families in both conditions attended a session together (DDDG: 45.2%; SET: 51.3%), however there was also a large proportion of families in which only the mother attended and had direct contact with the intervention material (DDDG: 41.9%; SET: 25.6%).

### Mother Short-Term Effects

#### Child Outcomes

Table 2 presents the descriptive statistics, univariate *F* values, relative effects sizes, and 95% confidence intervals for the short-term condition effects for mothers. A significant multivariate condition effect was observed for disruptive child behavior, *F*(2, 70) = 6.94, *p* = 0.002. Medium sized univariate condition effects were found for both the ECBI Intensity (*d* = 0.54) and ECBI Problem subscales (*d* = 0.65). Mothers in the SET condition reported a significantly lower number and frequency of disruptive child behaviors at post-intervention when compared to mothers in the DDDG condition. The MANCOVA for target and non-target negative child behaviors (PDR) and the ANCOVA for child psychosocial problems (SDQ) did not show significant condition effects for mothers at post-intervention.

**Table 2:** Descriptive statistics and univariate condition effects for mothers at post-intervention and 6-month follow-up

Measure	DDDG (*N* = 34)			SET (*N* = 41)			Condition effects					
	Pre-intervention	Post-intervention	6-month follow-up	Pre-intervention	Post-intervention	6-month follow-up	Post- intervention			6-month follow-up		
	M (SD)	M (SD)	M (SD)	M (SD)	M (SD)	M (SD)	F	p	d (95% CI)	F	p	d (95% CI)
Child outcomes												
ECBI intensity	150.10 (30.51)	133.49 (27.01)	131.46 (33.23)	145.53 (22.24)	114.50 (19.25)	115.75 (24.22)	13.45	**0.001**	0.54 (0.08, 1.00)	4.95	**0.031**	0.42 (− 0.04, 0.87)
ECBI problem	19.37 (6.23)	15.96 (7.32)	15.48 (8.17)	19.40 (5.55)	12.14 (5.66)	11.88 (6.67)	9.17	**0.004**	0.65 (0.19, 1.11)	4.73	**0.034**	0.61 (0.15, 1.07)
SDQ total difficulties	15.16 (5.89)	13.60 (5.92)	12.72 (7.10)	15.11 (5.94)	12.26 (4.91)	11.80 (5.87)	1.83	0.185	0.22 (− 0.24, 0.67)	0.64	0.460	0.15 (− 0.31, 0.60)
PDR weekday^a^	11.46 (5.39)	9.68 (4.66)	10.18 (5.07)	10.40 (6.44)	7.87 (3.77)	7.56 (3.99)	2.55	0.116	0.12 (− 0.33, 0.58)	4.67	0.039	0.26 (− 0.20, 0.71)
PDR weekend day^b^	12.71 (5.41)	8.90 (4.55)	9.48 (5.26)	10.92 (5.81)	6.76 (4.28)	6.72 (3.97)	2.53	0.117	0.06 (− 0.40, 0.52)	4.38	0.046	0.17 (− 0.29, 0.63)
Parenting outcomes												
PS	3.45 (0.58)	3.11 (0.56)	3.07 (0.49)	3.49 (0.61)	2.80 (0.61)	2.79 (0.60)	7.57	**0.008**	0.58 (0.12, 1.04)	5.50	**0.023**	0.53 (0.07, 0.99)
PTC behavioral	69.33 (22.97)	76.49 (15.96)	78.90 (15.31)	64.06 (20.07)	80.35 (14.42)	85.67 (10.75)	4.84	**0.031**	0.42 (− 0.03, 0.88)	8.62	**0.004**	0.56 (0.10, 1.01)
PTC setting	81.27 (14.93)	88.30 (8.49)	87.34 (10.30)	80.80 (12.97)	88.78 (9.02)	92.55 (6.17)	0.20	0.662	0.07 (− 0.38, 0.52)	9.54	**0.003**	0.40 (− 0.05, 0.86)
PES parenting experience	14.35 (3.64)	16.34 (3.15)	16.09 (4.14)	15.07 (2.79)	17.40 (2.62)	17.49 (2.67)	1.65	0.216	0.11 (− 0.35, 0.56)	2.25	0.163	0.21 (− 0.24, 0.66)
PPC extent^c^	33.22 (15.11)	29.03 (14.22)	33.24 (16.03)	38.57 (17.67)	34.02 (17.66)	32.09 (16.33)	0.30	0.598	0.02 (− 0.46, 0.50)	1.34	0.256	0.39 (− 0.10, 0.88)
PPC problem^c^	5.73 (3.93)	3.97 (3.58)	4.86 (3.48)	6.32 (4.06)	5.28 (3.93)	4.25 (3.57)	1.68	0.223	− 0.18 (− 0.66, 0.31)	2.51	0.125	0.30 (− 0.19, 0.78)
Mental health												
DASS− 21	10.85 (8.21)	7.69 (5.95)	9.47 (8.25)	10.23 (8.07)	7.28 (5.77)	6.28 (4.54)	0.03	0.877	− 0.03 (− 0.48, 0.42)	4.90	**0.031**	0.31 (− 0.14, 0.77)
Partner relationship												
PES partner support^d^	10.36 (2.51)	10.97 (1.60)	10.43 (2.38)	9.60 (3.10)	10.74 (2.69)	11.05 (2.96)	0.42	0.531	0.18 (− 0.31, 0.68)	5.24	**0.036**	0.48 (− 0.01, 0.97)
RQI^e^	32.32 (9.29)	34.50 (6.25)	31.44 (9.89)	32.09 (10.20)	33.55 (9.40)	34.80 (9.11)	0.48	0.521	− 0.07 (− 0.56, 0.41)	4.66	0.059	0.36 (− 0.13, 0.85)

*F* = univariate effect for condition, significant *p* values are bolded; *d* = effect size for condition; *d* 0.20 ≤ 0.49 = small, *d* 0.50 ≤ 0.79 = medium, *d* ≥ 0.80 = large

^a^*n* = 33 for the DDDG condition, *n* = 41 for the SET condition

^b^*n* = 33 for the DDDG condition, *n* = 39–40 for the SET condition

^c^*n* = 29 for the DDDG condition, *n* = 36 for the SET condition

^d^*n* = 28–29 for the DDDG condition, *n* = 35 for the SET condition

^e^*n* = 29 for the DDDG condition, *n* = 35 for the SET condition

#### Parenting Outcomes

The ANCOVAs for ineffective parenting behavior and behavioral parenting self-efficacy showed significant condition effects. At post-intervention, mothers in the SET condition reported fewer ineffective parenting behaviors and greater parenting self-efficacy in handling difficult child behaviors when compared to mothers in the DDDG condition. The condition effect was medium in size for parenting behaviors (*d* = 0.58) and small in size for behavioral parenting self-efficacy (*d* = 0.42). There were no short-term condition effects on parenting self-efficacy across settings, parenting experiences, or inter-parental conflict.

#### Mental Health and Partner Relationship

There were no significant condition effects on mothers’ mental health or their perceptions of partner support and partner relationship satisfaction at post-intervention.

### Father Short-Term Effects

#### Child Outcomes

Table 3 displays the descriptive statistics, univariate *F* values, *d* values, and 95% confidence intervals for fathers’ short-term outcomes. The MANCOVA for disruptive child behavior did not reveal any significant multivariate condition effects at post-intervention for fathers, however medium effects in favour of the SET condition over the DDDG condition were found on the ECBI Problem subscale (*d* = 0.73). There was, however, a significant multivariate effect on target and non-target negative child behaviors for fathers, *F*(2, 51) = 3.55, *p* = 0.038. The univariate condition effect indicated that fathers in the SET condition reported less target and non-target negative child behaviors on weekdays (*d* = 0.79), but not weekend days, than fathers in the DDDG condition. No condition effect was found for father-rated child psychosocial problems on the SDQ at post-intervention.

**Table 3:** Descriptive statistics and univariate condition effects for fathers at post-intervention and 6-month follow-up

Measure	DDDG (*N* = 27)			SET (*N* = 31)			Condition effects					
	Pre-intervention	Post-intervention	6-month follow-up	Pre-intervention	Post-intervention	6-month follow-up	Post-intervention			6-month follow-up		
	M (SD)	M (SD)	M (SD)	M (SD)	M (SD)	M (SD)	F	p	d (95% CI)	F	p	d (95% CI)
Child outcomes												
ECBI intensity	134.89 (26.89)	118.59 (23.62)	113.51 (17.22)	142.81 (29.87)	114.48 (27.16)	113.51 (25.83)	1.03	0.320	0.42 (− 0.10, 0.93)	0.12	0.742	0.27 (− 0.24, 0.79)
ECBI problem	16.37 (6.64)	14.93 (7.17)	12.70 (5.39)	18.14 (7.80)	11.28 (6.51)	10.29 (7.82)	5.89	0.019	0.73 (0.21, 1.26)	2.81	0.107	0.57 (0.05, 1.09)
SDQ total difficulties	12.52 (5.24)	11.15 (4.65)	10.68 (3.49)	14.79 (6.21)	12.49 (4.84)	12.31 (5.50)	0.62	0.461	0.16 (− 0.35, 0.67)	0.51	0.501	0.11 (− 0.40, 0.62)
PDR weekday^a^	8.73 (4.81)	9.38 (4.29)	8.24 (4.05)	10.90 (6.83)	6.70 (3.92)	6.16 (3.83)	7.23	**0.010**	0.79 (0.26, 1.33)	5.62	0.025	0.70 (0.16, 1.23)
PDR weekend day^b^	8.78 (4.72)	8.98 (5.15)	8.33 (4.12)	10.17 (6.58)	7.55 (4.16)	6.81 (3.62)	1.88	0.178	0.48 (− 0.04, 1.00)	3.35	0.076	0.49 (− 0.03, 1.02)
Parenting outcomes												
PS total	3.33 (0.54)	2.96 (0.55)	2.99 (0.64)	3.32 (0.73)	2.83 (0.72)	2.78 (0.63)	0.85	0.373	0.18 (− 0.33, 0.69)	2.10	0.159	0.30 (− 0.21, 0.82)
PTC behavioral	74.28 (21.08)	80.38 (12.94)	83.72 (11.20)	68.38 (22.30)	82.90 (12.91)	85.04 (12.44)	1.76	0.190	0.38 (− 0.13, 0.90)	1.32	0.258	0.33 (− 0.18, 0.84)
PTC setting	86.84 (9.45)	90.03 (6.84)	89.81 (6.82)	82.50 (11.65)	86.99 (9.05)	90.17 (9.01)	0.69	0.413	0.12 (− 0.39, 0.63)	2.36	0.131	0.43 (− 0.08, 0.95)
PES parenting experience	17.07 (3.85)	18.36 (2.34)	18.15 (2.77)	15.81 (3.39)	18.03 (2.81)	18.00 (2.19)	0.06	0.847	0.25 (− 0.26, 0.77)	0.71	0.483	0.30 (− 0.21, 0.82)
PPC extent^b^	32.19 (16.19)	28.21 (12.06)	26.23 (8.28)	38.19 (18.92)	30.33 (14.05)	34.14 (15.43)	0.02	0.885	0.22 (− 0.30, 0.73)	3.85	0.055	− 0.11 (− 0.62, 0.41)
PPC problem^b^	5.35 (3.90)	4.43 (3.37)	3.52 (2.81)	5.97 (4.13)	4.63 (3.51)	4.80 (3.67)	0.04	0.866	0.10 (− 0.41, 0.62)	1.79	0.204	− 0.16 (− 0.68, 0.35)
Mental health												
DASS− 21	7.59 (6.79)	7.44 (5.90)	7.37 (4.97)	11.73 (10.10)	8.72 (7.95)	9.73 (9.19)	0.12	0.796	0.32 (− 0.19, 0.84)	0.19	0.679	0.20 (− 0.31, 0.71)
Partner relationship												
PES partner support^c^	10.74 (2.70)	11.78 (1.69)	11.90 (1.68)	10.53 (2.49)	11.04 (2.61)	11.24 (2.59)	1.60	0.222	− 0.20 (− 0.72, 0.32)	1.38	0.285	− 0.17 (− 0.69, 0.34)
RQI^b^	33.62 (8.47)	36.25 (5.39)	35.01 (6.23)	33.41 (8.69)	34.46 (8.28)	33.19 (9.84)	1.22	0.303	− 0.18 (− 0.70, 0.33)	0.75	0.412	− 0.18 (− 0.70, 0.33)

*F* = univariate effect for condition, significant *p* values are bolded; *d* = effect size for condition; *d* 0.20 ≤ 0.49 = small, *d* 0.50 ≤ 0.79 = medium, *d* ≥ 0.80 = large

^a^*n* = 25 for the DDDG condition, *n* = 31 for the SET condition

^b^*n* = 26 for the DDDG condition, *n* = 31 for the SET condition

^c^*n* = 26 for the DDDG condition, *n* = 30–31 for the SET condition

#### Parenting, Mental Health and Partner Relationship Outcomes

There were no short-term condition effects on the parenting measures for fathers, nor were there any condition effects for fathers’ mental health, fathers’ perceptions of support from their partner, or relationship satisfaction at post-intervention.

### Mother Maintenance Effects

#### Child Outcomes

Significant univariate condition effects for disruptive child behavior were maintained at 6-month follow-up (see Table 2). Mothers in the SET condition continued to report a lower frequency (*d* = 0.42) and number (*d* = 0.61) of disruptive child behaviors when compared to mothers in the DDDG condition. No significant differences in mother-rated child psychosocial problems and target and non-target negative child behavior were found at 6-month follow-up.

#### Parenting Outcomes

Significant condition effects for parenting behavior and behavioral parenting self-efficacy found at post-intervention were maintained at 6-month follow-up (*d*’s were 0.53 and 0.56 respectively). In addition, a small univariate condition effect was found for mothers’ setting parenting self-efficacy at 6-month follow-up (*d* = 0.40), with mothers in the SET condition reporting higher parenting self-efficacy across a range of settings than mothers in the DDDG condition. As at post-intervention, no condition effects for mother-rated parenting experiences and inter-parental conflict were found at 6-month follow-up.

#### Mental Health and Partner Relationship

Significant univariate condition effects were found for mental health (*d* = 0.31) and perceptions of support from their partner (*d* = 0.48) at 6-month follow-up. Mothers in the SET condition reported better mental health and more positive perceptions of partner support than mothers in the DDDG condition. No condition effect was found for partner relationship satisfaction at 6-month follow-up for mothers.

### Father Maintenance Effects

The MANCOVAs and ANCOVAs examining maintenance condition effects for fathers did not reveal any significant condition effects on any child or parent outcome measure (see Table 3) indicating that effects found at post-intervention were not maintained at 6-month follow-up.

### Completer Analyses

The MANCOVAs and ANCOVAs examining the short-term and maintenance condition effects were repeated using only the sample of mothers and fathers who completed outcome measures at post-intervention and 6-month follow-up (see Fig. 1). Among the completer sample, the short-term condition effects for mothers were still significant and effect sizes were similar for disruptive child behaviour (ECBI Intensity: *d* = 0.38, 95% CI − 0.12, 0.88; ECBI Problem: *d* = 0.62, 95% CI 0.10, 1.13) and parenting behavior (PS: *d* = 0.60, 95% CI 0.09, 1.11), but the condition effect for behavioral parenting self-efficacy (PTC) was no longer significant, (*d* = 0.30, 95% CI − 0.21, 0.81). The effect sizes for mother-rated disruptive child behavior, parenting behavior, parenting self-efficacy, mental health, and perceptions of partner support found among the completer sample at 6-month follow-up were similar in size to the ITT sample, even though the condition effects were not significant (completer sample: ECBI Intensity: *d* = 0.19, 95% CI − 0.33, 0.71; ECBI Problem: *d* = 0.68, 95% CI 0.14, 1.21; PS: *d* = 0.54, 95% CI 0.02, 1.07; PTC Behavior: *d* = 0.35, 95% CI − 0.17, 0.88; PTC Setting: *d* = 0.21, 95% CI − 0.31, 0.73; DASS-21: *d* = 0.11, 95% CI − 0.43, 0.65; PES Partner Support: *d* = 0.44, 95% CI − 0.10, 0.97). For fathers, all significant condition effects found in the ITT sample were also found in the completer sample, with a large effect found for PDR Weekday at post-intervention (*d* = 0.99, 95% CI 0.37, 1.60). In addition, a significant multivariate condition effect for disruptive child behavior was found at post-intervention, *F*(2, 42) = 4.16, *p* = 0.023. Univariate analyses showed that the effect between conditions was found on the ECBI Problem Total subscale only, with fathers in the SET condition reporting less child disruptive behaviors at post-intervention than fathers in the DDDG condition. The size of the condition effect was large (*d* = 1.05, 95% CI 0.45, 1.66).

### Statistically Reliable and Clinically Significant Change

Significantly more mothers in the SET condition reported pre- to post-intervention improvements in the frequency of their child’s disruptive behavior that were statistically reliable and clinically significant than those in the DDDG condition (see Table 4). Among fathers, a significantly greater proportion of those in the SET condition reported statistically reliable improvement and clinically significant improvement in the number of disruptive behaviors displayed by their child when compared with fathers in the DDDG condition. A small proportion of parents in each condition (*n* = 1–3) reported deterioration in their child’s disruptive behavior between pre- and post-intervention.

**Table 4:** Statistically reliable and clinically significant change from pre- to post-intervention on the ECBI by condition

Measure	Mothers						Fathers					
	DDDG (N = 34)		SET (N = 41)		Fisher’s χ^2^	p	DDDG (n = 27)		SET (n = 31)		Fisher’s χ^2^	p
	n	%	n	%			n	%	N	%		
Statistically reliable change												
ECBI intensity					6.96	**0.021**					1.79	0.461
Reliably improved	11	32.4	26	63.4			10	37.0	16	51.6		
Reliably deteriorated	3	8.8	1	2.4			2	7.4	2	6.5		
No reliable change	20	58.8	14	34.1			15	55.6	13	41.9		
ECBI problem					5.41	0.051					7.87	**0.015**
Reliably improved	10	29.4	21	51.2			4	14.8	15	48.4		
Reliably deteriorated	2	5.9	0	0.0			2	7.4	2	6.5		
No reliable change	22	64.7	20	48.8			21	77.8	14	45.2		
Clinically significant change												
ECBI intensity					11.05	**0.009**					3.32	0.339
Clinically significant change	8	23.5	23	56.1			8	29.6	15	48.4		
Did not achieve clinical change	14	41.2	7	17.1			6	22.2	6	19.4		
Worsened	2	5.9	0	0.0			1	3.7	0	0.0		
Not in clinical range	10	29.4	11	26.8			12	44.4	10	32.3		
ECBI problem					7.50	0.055					8.35	**0.041**
Clinically significant change	9	26.5	23	56.1			5	18.5	15	48.4		
Did not achieve clinical change	18	52.9	12	29.3			12	44.4	6	19.4		
Worsened	2	5.9	1	2.4			2	7.4	2	6.5		
Not in clinical range	5	14.7	5	12.2			8	29.6	8	25.8		

Significant *p* values are bolded

### Participant Satisfaction

Overall satisfaction with each of the Triple P Discussion Groups was relatively high (*M*’s ranged from 49.98 to 55.52). A high proportion of parents rated the quality of the groups as at least ‘good’ (range 77.0–96.0%). Many reported that they had gained sufficient knowledge to be able to implement the parenting strategies introduced in session (range 84.5–100.0%). A high level of intent to use the strategies was also indicated. Satisfaction with the format and content of the groups was also high, but satisfaction with the amount and type of help provided during each discussion group varied by topic with lower satisfaction reported for the Dealing with Disobedience group. The extent to which the groups met parents’ needs was also lowest for Dealing with Disobedience.

## Discussion

This study compared the effects of narrowly focused training and sufficient exemplar training of low-intensity topic-specific parenting groups on a range of child and parent outcomes using an RCT design. The effects for both mothers and fathers of children displaying at least mild conduct problems were examined. The results partially supported our hypothesis that SET would lead to better intervention outcomes for children. At post-intervention, mothers in the SET condition reported a lower frequency and number of disruptive child behaviors, the primary outcome measure for the study, and fathers reported that their child displayed less target and non-target negative behaviors on weekdays. The relative effect sizes showed there were medium effects for sufficient exemplars over and above the narrowly focused training condition. Lower levels of mother-rated disruptive child behavior was maintained at 6-month follow-up. In addition, when compared to parents in the DDDG condition, a greater proportion of mothers and fathers in the SET condition reported statistically reliable and clinically significant reductions in their child’s disruptive behavior from pre- to post-intervention. Greater change in child behavior among the SET condition was assumed to be a result of the generalization promotion strategy that aimed to enhance parents’ ability to apply parenting skills flexibly and feel more confident in their parenting, resulting in a broader, more robust change in child behavior.

Even though the results overall indicated that mothers and fathers in the SET condition reported greater improvement on their child’s disruptive behavior, the primary outcome measure for the study, significant condition effects did not emerge across all secondary child outcome measures at all time points. A possible explanation for this is that because families were screened into the study based on reporting elevated disruptive child behavior problems using an abbreviated version of the ECBI, there was sufficient scope for change on this measure. Whereas the SDQ scores for mothers and fathers were in the borderline range at pre-intervention, and even though on average they moved into the normal range at post-intervention, there was less scope for improvement. Furthermore, the PDR may be less sensitive to change given that it provides a measure of the occurrence and non-occurrence of negative child behaviors on a specific day, although a medium effect was found for fathers at 6-month follow-up on the PDR Weekday subscale.

There was support for the hypothesis that SET would lead to better intervention outcomes for parents. Mothers in the SET condition reported fewer ineffective parenting behaviors and greater parenting self-efficacy across a range of behaviors at post-intervention. These condition effects were maintained at 6-month follow-up. Furthermore, at 6-month follow-up, mothers in SET condition also reported greater parenting self-efficacy for dealing with difficult child behaviors in a range of settings, less mental health difficulties, and more positive perceptions of support from their partners in comparison to mothers in the DDDG condition. The effect sizes for these measures were small to medium in size. Additional benefits for mothers demonstrated in the study further supports that the generalization of parenting skills promoted by SET led to superior intervention outcomes. However, it should be acknowledged that the study results might be partly explained by participants in the SET condition finding the topics more relevant to them than the topic of the narrowly focused training condition. Support for this possibility is provided by the lower satisfaction ratings for the disobedience topic relative to the other groups.

In contrast to mothers, SET did not result in greater intervention effects for fathers on measures of parenting, mental health, or partner relationship at post-intervention or 6-month follow-up. It is unclear if these results represent a real lack of effect. There are several alternative explanations of the outcomes for fathers in the SET condition. Less positive results for fathers may be a result of lower attendance of fathers in the interventions. Among families in the SET condition, a greater number of fathers than mothers did not attend any of the sessions. Furthermore, a substantial proportion of mothers from two-parent families attended alone. Taken together, these findings indicate that fathers had less direct contact with the intervention material and thus had fewer opportunities than mothers to learn and practice parenting strategies.

Previous research has reported that attending a DDDG leads to positive outcomes for parents and their young children [16, 18]. The findings from the current study add to this literature by indicating that multiple training exemplars of four TPDGs produces greater change for mothers and young school-aged children. The previous evaluations of the DDDG have also reported high satisfaction among parents of primarily preschool aged children. In the current study, the TPDGs were generally acceptable to parents of young school-aged children. Parents indicated that overall the sessions were relevant and useful.

There are several implications for practice that arose from the current study. Results demonstrated that SET of low-intensity topic-specific parenting groups appears to have additional benefits for improving mother- and father-rated child behavior and mothers’ parenting behavior, parenting self-efficacy, mental health, and perceptions of partner support. For families with young children displaying mild to moderate conduct problems, practitioners could consider teaching parenting skills through training in a sufficient number of exemplars. Narrowly focused training could be a first line of approach to intervention with training in additional exemplars reserved for those who fail to generalize parenting skills effectively. More intensive parenting programs could then be reserved for families who do not benefit from training in several exemplars, those with children displaying high levels of conduct problems, and those with multiple family risk factors.

A key aim of low-intensity programs is to improve the cost-effectiveness of interventions [11] and consideration needs to be given to the cost of additional sessions, the potential added benefits for children and parents, the increased risk of attrition with a greater number of sessions, and the feasibility within a population health approach to parenting support. Delivering multiple exemplars adds to the cost of the intervention and requires more time from parents but increases effect sizes of low-intensity parenting groups. Parents’ needs and preferences for parenting support would also likely influence the uptake of sufficient exemplar training. It may be that parents are more likely to take part in several topic-specific parenting groups that are tailored to their particular parenting challenges than a more general parenting program. In addition, multiple groups enable greater opportunity for more than one parent to participate, either separately or together.

Low-intensity parenting groups may also be a way to engage and enhance father participation in parenting programs. Among two-parent families, both parents should be encouraged to attend and engage with the program to promote co-parenting [19]. Child care services could be offered to enable mothers and fathers to participate in parenting programs. Flexible delivery options timed to suit both parents could include offering evening sessions or full-day weekend workshops in easy to access settings. Ways to enhance father participation in low-intensity topic-specific parenting groups should be investigated as such programs appear to be an attractive option for intervention among fathers.

While the self-referral method for recruitment used is a clinically viable method which could be undertaken in settings that do not have substantial budgets or resources [36], it may result in samples that are not representative of the general population of families with similar problems. In relation to this point, although attrition from the study was relatively low, there were more single parent families, more mothers with lower levels of education, and more low SES families among those who did not complete post-intervention measures. If additional resources had been available, further efforts could have been made to retain these participants and sample across a more diverse range of socioeconomic backgrounds and promote participation among ethnic minorities.

Furthermore, the study relied on self-report measures to evaluate the intervention outcomes. It is unknown whether changes reported on the outcome measures were actually observed or whether improvements relate to changes in parents’ perceptions of their child and parenting. However, parental reports are particularly valuable given their unique knowledge about their child’s behavior and their status as participants [37], and in the current study information on child behavior was sought from both parents. As observational measures can be subject to reactivity effects and may inadequately measure low prevalence behaviors, ideally, intervention outcomes and the generalization of parenting skills should be measured using both self-report measures and observational methods. The current study was unable to obtain observational measures due to budget constraints.

Further trials with larger samples, more diverse families, and longer-term follow-up would extend the findings from the current study. It remains an empirical question as to how many additional exemplars are required before superior intervention outcomes are attained, and whether this number is the same for mothers and fathers and those parenting as a couple or by themselves. It is possible that additional benefits may have been found after two or three exemplars; thus, future research could investigate this possibility. Future research should also aim to directly compare the effects of receiving SET of topic-specific low-intensity parenting groups with a high-intensity group parenting program as well as examine moderators or predictors of intervention outcomes.

Overall, low-intensity parenting groups that are topic-specific appear to be an acceptable option for intervention for parents with young school-aged children displaying conduct problems. The current study highlighted the potential of teaching using generalization promotion strategies to enhance child and maternal intervention outcomes of low-intensity topic-specific parenting groups.

## Summary

A large proportion of young children display mild to moderate levels of conduct problems. Survey data also indicates a high prevalence of ineffective parenting practices are used by parents of young children, suggesting a need for efficacious, cost-effective interventions. Low-intensity parenting groups, such as the Triple P-Positive Parenting Program Discussion Groups, appear to be a cost-effective intervention for child conduct problems. Several studies evaluating a Triple P Discussion Group on disobedience found promising results for improving child and parent outcomes. However, a sufficient exemplar training approach that incorporates generalization promotion strategies may assist parents to more flexibly apply positive parenting principles to a broader range of child target behaviors and settings, leading to greater change. We compared the effects of sufficient exemplar training to an existing narrowly focused low-intensity intervention. We predicted that this kind of training would help consolidate the learning of these skills and result in more robust intervention effects across a broader range of child and parent outcomes. We also predicted that sufficient exemplar training would lead to more robust changes at multiple levels of the family system, such as parental mental health and their partner relationship, than narrowly focused training. In addition, this study also targeted a research gap on the effects of low-intensity parenting programs for fathers, by attempting to engage fathers as well as mothers. Another focus of the study was to examine the effects of the Triple P Discussion Groups among parents with young school-aged children by addressing key topics relevant to this developmental phase.

Participants were 75 mothers and 58 fathers from 78 families with a 5–8 year old child residing in Auckland, New Zealand. A 2 (condition: narrowly focused training vs. sufficient exemplar training) by 3 (time: pre-intervention, post-intervention, 6-month follow-up) RCT design was used to compare the effects of the conditions on a range of child and parent outcomes.

We found that sufficient exemplar training resulted in more robust changes in child behavior and superior outcomes for mothers on measures of parenting behavior, parenting self-efficacy, mental health, and perceptions of partner support at post-intervention and 6-month follow-up. There was some support in favour of sufficient exemplar training for father-reported child behavior. However, in contrast to mothers, sufficient exemplar training did not result in greater intervention effects for fathers on measures of parenting, mental health, or partner relationship at post-intervention or 6-month follow-up. Attendance and overall satisfaction were generally high.

Topic-specific low-intensity parenting groups appear to be an acceptable option for intervention for parents with young school-aged children displaying conduct problems and may be a way to engage and enhance father participation in parenting programs. These results indicate that teaching sufficient exemplars may promote generalization leading to enhanced intervention outcomes.
